# Supplementary material for: Modulation of telomere protection by the PI3K/AKT pathway
Source: Nat Commun. 2017 Nov 2;8:1278. doi: 10.1038/s41467-017-01329-2 (PMC5668434; doi:10.1038/s41467-017-01329-2)
Supplement: Supplementary file 1 — Supplementary Information [file 41467_2017_1329_MOESM1_ESM.pdf]

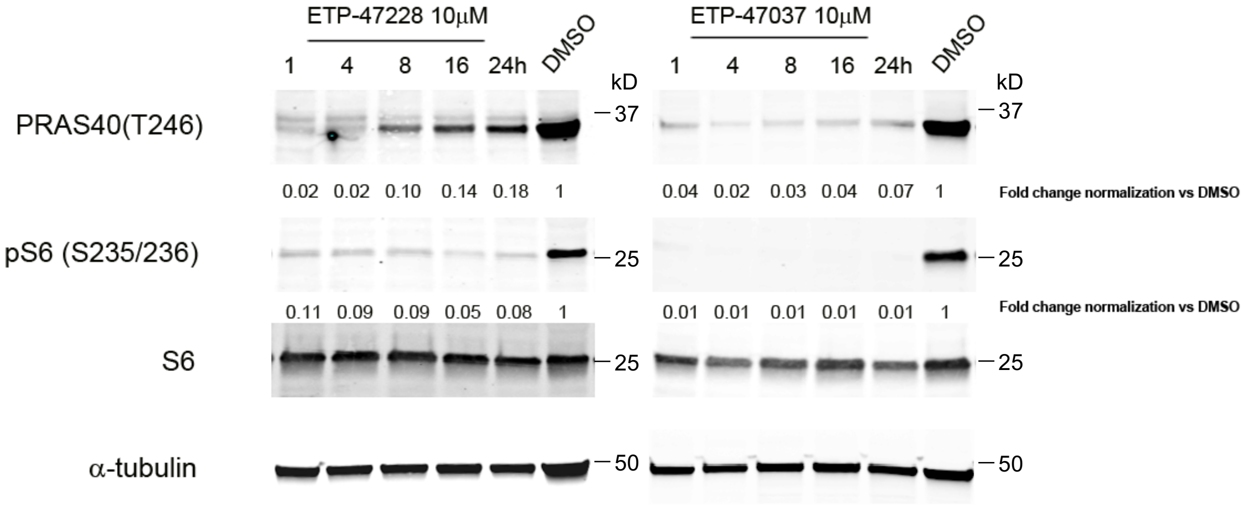

Supplementary Figure 1. Modulation of PI3K/AKT downstream targets by ETP-47037 and ETP-47228. Representative Western blot images of PRAS40 phosphorylation at T246 and of P6 at S235/236 in CHIA 9-3 lung cancer cell line treated for 24 hours with 10 μM of ETP-47037 or with 10 μM of ETP-47228 at different time points of the treatment. Ratio values PRAS40(T246)/Total tubulin and pS6/Total S6 are shown.

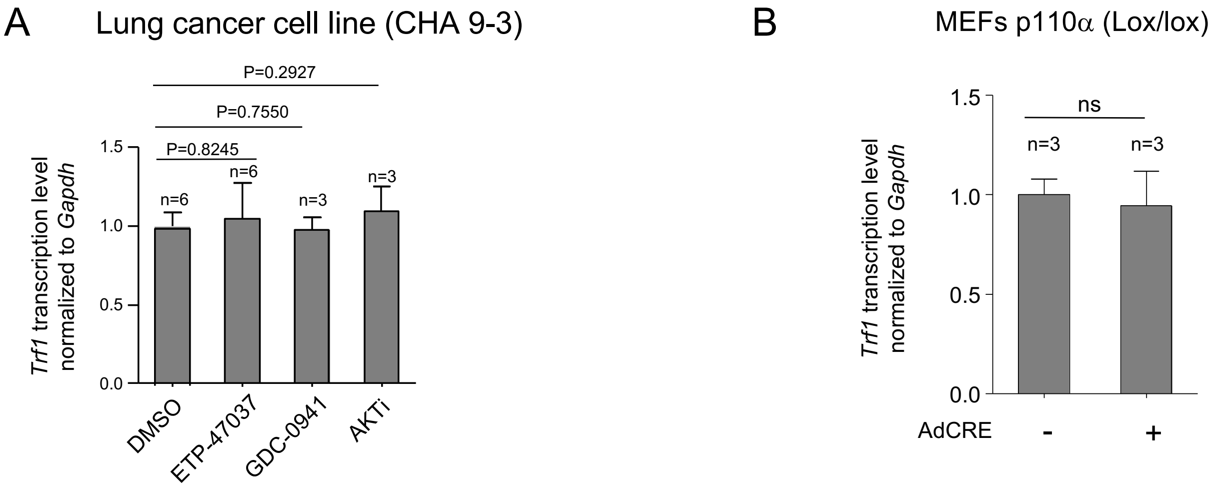

Supplementary Figure 2: Transcriptional TRF1 levels are not affected by PI3K/AKT inhibitors. (A) Quantification of TRF1 mRNA by qPCR in CHA 9-3 treated either with DMSO, PI3K inhibitors (ETP-47037 and GDC-941) and AKT inhibitor (AKTi) at 10μM 24h. **(B)** Quantification of TRF1 mRNA by qPCR in immortalized P110$\alpha^{lox/lox}$ MEFs either transduced with GFP or with Cre recombinase. Error bars represent standard deviation. The Student´s t-test was used for statistical analysis in each case and P values (P) are shown for each analysis. (n) Number of experiment per condition.

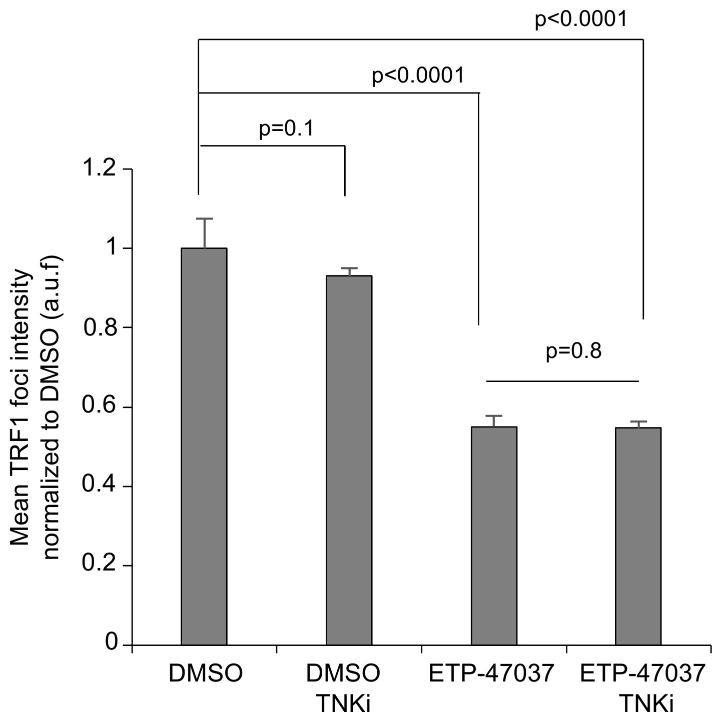

Supplementary Figure 3. Effects of Tankyrase inhibition on TRF1 downregulation by PI3K inhibitor EP-47037. Quantification of TRF1 immunofluorescence in lung cancer derived cells treated with DMSO and with 10μM ETP-47037, with or without 10μM Tankyrase inhibitor for 24h. Error bars represent standard deviation. The Student´s t-test was used for statistical analysis in each case and P values (P) are shown for each analysis.

Fig. 1C

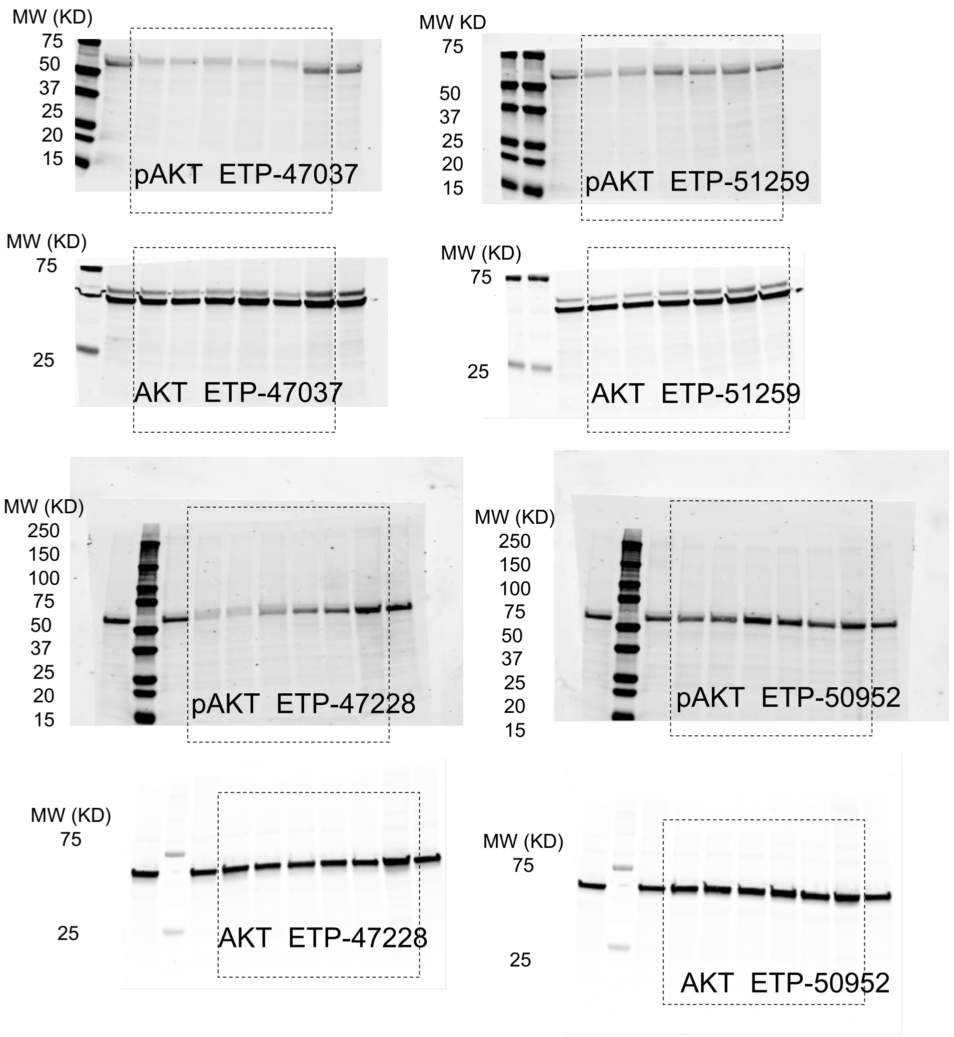

Fig. 2C

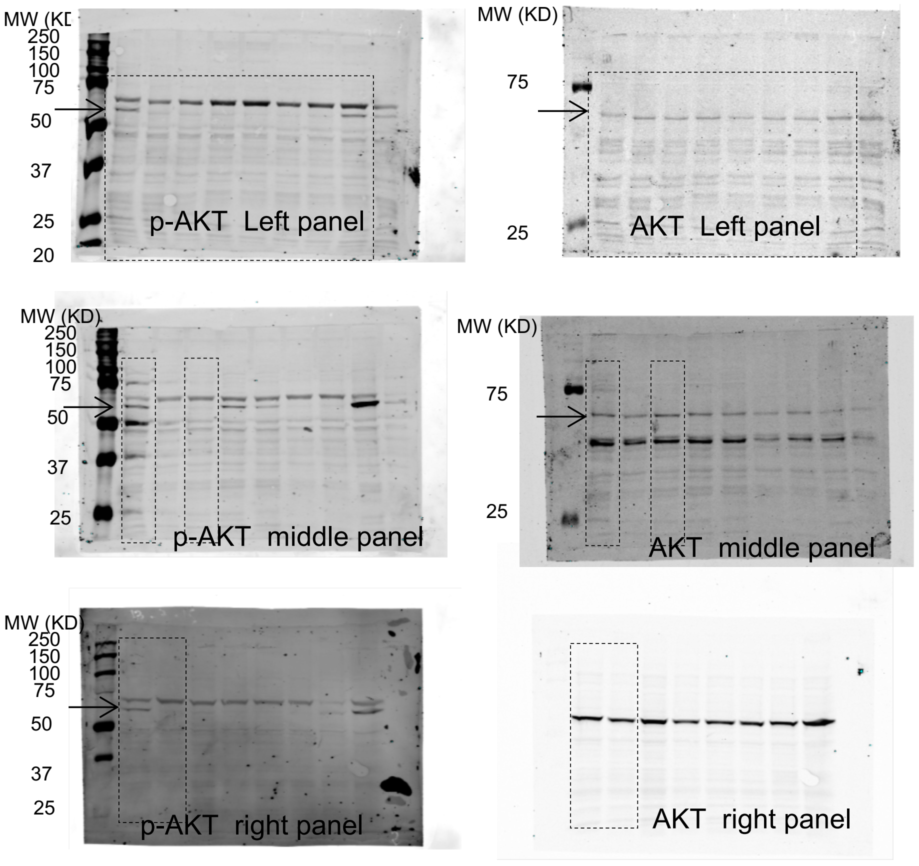

Supplementary Figure 4: Uncropped scans of the western blots presented in main Figures as indicated

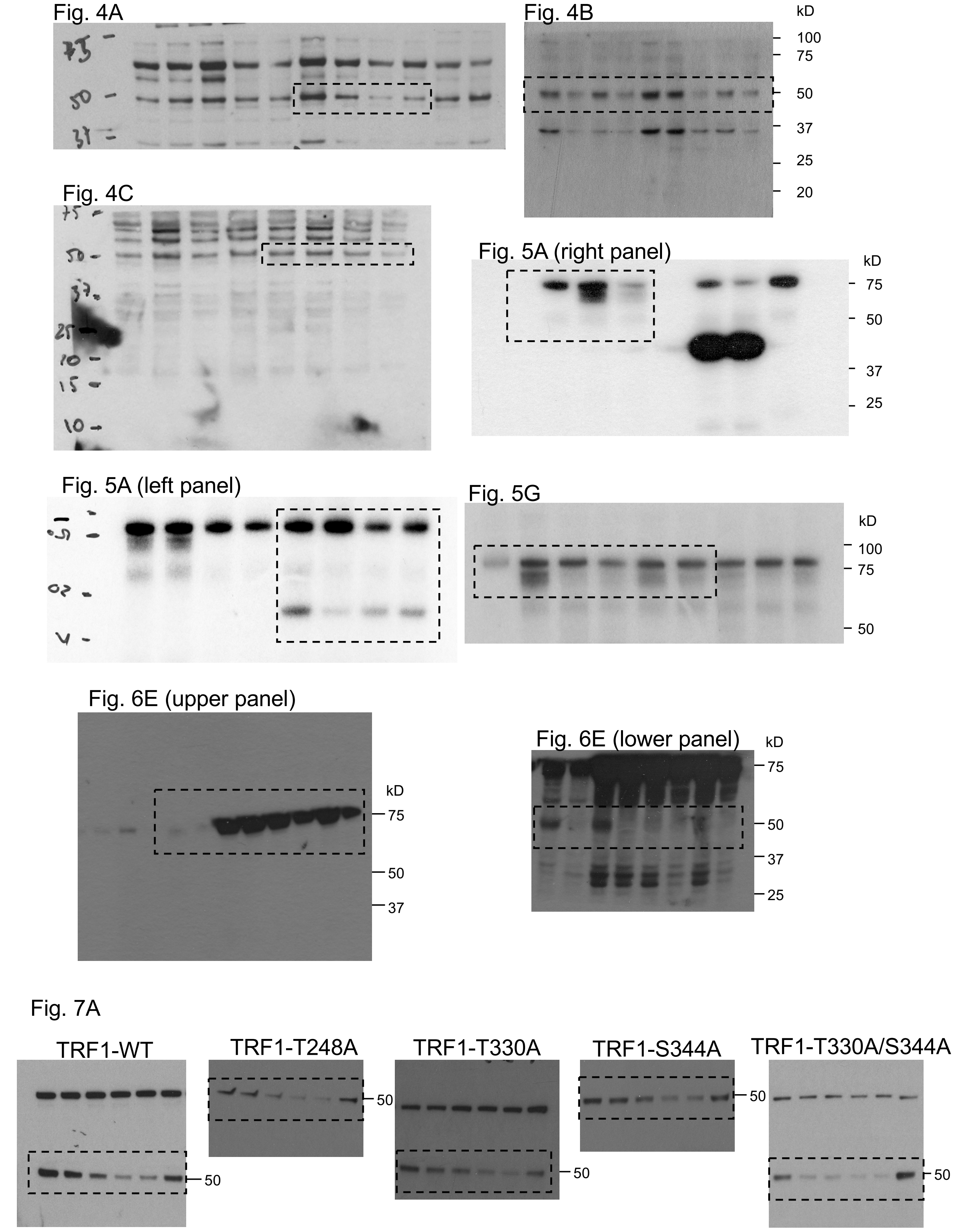

Supplementary Figure 5: Uncropped scans of the western blots presented in main Figures as indicated

Supplementary Table 1: Kinase selectivity profile of ETP-47037 and ETP-47228 at 1 μM. Selectivity against protein kinases was determined from evaluation of compounds at 1μM in ProQinase's 24 selected panel that represents the ACG, STE, TKL, TK, CMGC CAMK and CK1 kinase families.

	ETP-47037 Inh (%) at 1μM	ETP-47228 Inh (%) at 1μM
AKT2	-7,5	-8
B-RAF-V600E	6,5	11,5
CDK8-CycC	-4,5	1
CHK1	15,5	-11,5
CHK2	0,5	3
CK1-alpha1	-6	2
DYRK1A	-1	4
EGF-R	22,5	0,5
FAK	14,5	-7,5
FGF-R1	6,5	-3,5
IGF1-R	2	-19
IKK-beta	1	-10
INS-R	5	-3
JAK2	2	4
KIT	9	6,5
MEK1	-15	-3
MET	3	3
PAK1	-3,5	1
PDGFR-alpha	4	1
PDK1	7	-9
RPS6KA1	-4,5	-1,5
SGK1	0,5	0,5
SRC	26	5
VEGF-R2	21,5	-3
